# Prognostic significance of postoperative pneumonia after curative resection for patients with gastric cancer

**DOI:** 10.1002/cam4.1163

**Published:** 2017-10-26

**Authors:** Ru‐Hong Tu, Jian‐Xian Lin, Ping Li, Jian‐Wei Xie, Jia‐Bin Wang, Jun Lu, Qi‐Yue Chen, Long‐long Cao, Mi Lin, Chao‐Hui Zheng, Chang‐Ming Huang

**Affiliations:** ^1^ Department of Gastric Surgery Fujian Medical University Union Hospital Fuzhou China; ^2^ Department of General Surgery Fujian Medical University Union Hospital Fuzhou China; ^3^ Key Laboratory of Ministry of Education of Gastrointestinal Cancer Fujian Medical University Fuzhou China; ^4^ Fujian Key Laboratory of Tumor Microbiology Fujian Medical University Fuzhou China

**Keywords:** Complications, gastrectomy, prognosis, stomach cancer

## Abstract

Few studies have been designed to investigate the incidence of postoperative pneumonia after radical gastrectomy and its effect on prognosis of these patients. Incidences of postoperative pneumonia after radical gastrectomy in our department between January 1996 and December 2014 were summarized. Their effects on prognosis were retrospectively analyzed using survival curves and Cox regression. A total of 5237 patients were included in this study, 767 (14.4%) of them had complications, including 383 cases of postoperative pneumonia (7.2%). The 5‐year overall and disease‐specific survival of patients with postoperative pneumonia were both lower than those without this complication (*P *< 0.001). Stratified analysis demonstrated that this difference existed in all Stage I, II, and III patients (log‐rank, *P *< 0.05). Multivariate analysis revealed that age, neoadjuvant chemotherapy, tumor size, tumor stage, and postoperative pneumonia were independent risk factors for disease‐specific survival. Postoperative pneumonia after radical gastrectomy is an independent risk factor for prognosis of gastric cancer patients, especially in stage III.

## Introduction

Gastric cancer is one of the major threats to human health, ranking No. 4 among the most common cancerous diseases and No. 2 among the deadliest cancers 1. Despite some decrease in incidence and mortality in recent years, gastric cancer remains a great threat to human health. Surgical resection is the only possible cure for gastric cancer, which remains a need for methods to reduce the incidence of complications and to maximize the safety of the procedure while maintaining efficacy to eradicate cancer. Postoperative pneumonia is the most common complication after gastric resection, occurring in 1.1–12.32% of the patients 2, 3, 4, 5, 6, 7. Postoperative pneumonia causes additional pain for patients, prolongs their hospital stay, increases hospitalization costs, and even causes respiratory failure or early death. Recent studies suggest that postoperative pneumonia may even reduce long‐term survival 8, 9, 10, 11. Baba et al. 9 reported that postoperative pneumonia is an independent risk factor for disease‐specific survival after radical esophagectomy. Saeki et al. 10 believed that postoperative pneumonia and anastomotic fistulas reduced long‐term disease‐specific survival of esophageal squamous cell carcinoma patients. However, regarding the efficacy of gastric cancer surgery, although some studies have suggested a reduction in long‐term survival of gastric cancer patients due to postoperative complications 12, 13, 14, 15, no study has confirmed a correlation between postoperative pneumonia and prognosis after radical gastrectomy. Therefore, by retrospectively analyzing complications and prognoses of a large number of gastric cancer patients who received radical gastrectomy, this study intends to investigate the effect of postoperative pneumonia and treatment on long‐term survival of these patients.

## Materials and Methods

### Study subjects

This study was a retrospective analysis of prospectively collected data from a database of primary gastric cancer patients treated with radical gastrectomy in the Department of Gastric Surgery at Fujian Medical University Union Hospital in Fuzhou, China between January 1996 and December 2014. Gastric cancer staging was performed according to the 7th edition of the UICC TNM classification 16. Adjuvant chemotherapy was recommended for patients with advanced gastric cancer. Furthermore, after 2007, neoadjuvant chemotherapy was recommended for clinical stage III. Neoadjuvant chemotherapy and adjuvant chemotherapy were defined as at least one cycle of 5‐Fu‐based chemotherapy.

Patient inclusion criteria were as follows: a histologically confirmed adenocarcinoma of the stomach, no evidence of tumors invading adjacent organs (pancreas, spleen, liver, and transverse colon), para‐aortic lymph node enlargement, or distant metastasis demonstrated by abdominal computed tomography (CT) and/or abdominal ultrasound and posteroanterior chest radiographs, a D1+(D1 plus Nos. 8a, 9, 11p in total or proximal gastrectomy, or D1 plus Nos. 8a, 9 in distal gastrectomy)/D2 (D1 plus Nos. 8a, 9, 10, 11p, 11d, 12a in total gastrectomy, or D1 plus Nos. 8a, 9, 11p, 12a in distal gastrectomy) lymphadenectomy with digestive tract reconstruction, and a curative R0 according to postoperative pathological diagnosis. Patient exclusion criteria were as follows: intraoperative evidence of peritoneal dissemination or distant metastasis and incomplete pathological data. The extent of gastric resection and lymph node dissection was selected according to ver.1 and ver.3 of the Japanese gastric cancer treatment guidelines 17, 18.

### Variables and definitions

The definition of each complication was based on the literature 4, 9, 19, 20, 21, 22, 23, 24. Postoperative pneumonia was defined as a newly developed infiltrates on the chest radiograph and positive results of bronchoalveolar lavage culture 9, 19. We defined surgical site infections (SSIs) according to the surgical patient component of the 1999 Centers for Disease Control and Prevention (CDC) National Nosocomial Infection Surveillance (NNIS) System manual; this definition includes incisional (superficial, deep) and organ/space SSIs 20. Postoperative bleeding was defined as abdominal drainage or bloody nasogastric drainage, hematemesis, melena, decreased hemoglobin, unexplained hypotension or tachycardia, or a clear diagnosis by endoscopy, angiography, scintigraphy (radionuclide), CT, or reoperation when the patient's clinical condition deteriorated 21. Intestinal ileus suggested postoperative intestinal obstruction in abdominal X‐ray, as did isolated, fixed, and swollen intestinal loops or intestinal wall edema, thickening, adhesions, intestinal gas accumulation, uniform expansion of intestine, and peritoneal exudation in abdominal CT scan 22, 23. Remnant gastric stasis was defined as postprandial nausea, vomiting, and other symptoms of gastric stasis in patients without anastomotic stenosis, intestinal obstruction, and abdominal infection who still needed nasogastric tube decompression 4 days after surgery or who required gastric tube replacement 3 days after surgery. A chylous leak was defined as >200 mL/day milky white fluid that was positive in the chyluria test and a triglyceride level >110 mg/dL 24.

Complications were classified according to the modified version of the Clavien‐Dindo classification system reported by Dindo et al. 25. When calculated the postoperative complications rate, the most severe complication was noted in the cases in which more than one complication occurred in a patient. The study focuses on specific complication's effect on prognoses. So, we also count each type of complication no matter whether it is the most severe complication. Active intervention for postoperative pneumonia included ventilator‐assisted breathing, pleural aspiration and bronchoscopic aspiration, and other invasive operations.

### Follow‐up

The patients were followed up by outpatient or home visits, e‐mails, or phone calls with intervals of 3–6 months until March 2016 or the death of the patient, with a median follow‐up length of 35 months. Overall survival was defined as the interval between the date of the operation and the date of death. Disease‐specific survival was defined as the interval between the date of operation and the date of death that was confirmed to be attributable to gastric cancer.

### Statistical analyses

Continuous data were reported as mean ± SD and were analyzed using Student's *t*‐test. The categorical data were presented as proportion percentages and were analyzed using Pearson's chi‐square test or Fisher's exact test. Survival analysis was performed using the Kaplan–Meier method to assess survival time distribution and using the log‐rank test where indicated. Univariate and multivariate analyses with the Cox proportional hazard model were adopted to clarify the independent prognostic factors. Variables with *P *< 0.10 in the univariate analysis were subsequently included in a multivariate Cox regression model. Overall, a *P *< 0.05 was considered statistically significant. The statistical analyses were performed with the SPSS version 18.0 (SPSS, Chicago, IL) and R software (The R Project for Statistical Computing; The R Foundation, Vienna, Austria).

## Results

### Incidence of postoperative pneumonia

Between January 1996 and December 2014, 5327 patients who received radical resection for gastric cancer were included in the study. A total of 767 (14.4%) of these patients had postoperative complications, 26 (0.5%) of these patients died during the perioperative period, and 121 (2.3%) of these patients died within 90 days. The most common complication was pneumonia (7.2%, 383/5327), followed by SSIs (5.1%, 273/5327), postoperative bleeding (1.1%, 58/5327), intestinal ileus (1.1%, 56/5327), remnant gastric stasis (0.9%, 50/5327), and chylous leak (0.8%, 41/5327). The postoperative pneumonia cases included 283 Grade II cases (5.3%), 36 Grade III cases (0.7%), 39 Grade IVa cases (0.7%), 7 Grade IVb cases (0.1%), and 18 Grade V cases (complication‐related death, 0.3%). General clinical and pathological data of these patients are listed in Table 1. Postoperative pneumonia was closely correlated with age, gender, Charlson index, ASA score, tumor size, TNM stage, resection modality, resection range, operative time, and intraoperative blood loss (*P *< 0.05). Additionally, the rate of postoperative chemotherapy was lower in those with pulmonary complications than in those without (41.8% vs. 25.6%, respectively, *P *< 0.001). Multivariate analyses show that age (OR = 1.541, *P *< 0.001), ASA classification ≥3 (OR = 2.202, *P *< 0.001), Tumor diameter (OR = 1.068, *P* = 0.002), Open gastrectomy (OR = 1.458, *P* = 0.013), Operative time (OR = 1.119, *P* = 0.001), and Blood loss (OR = 1.105, *P *< 0.001) were independent risk factors for postoperative pneumonia (Table 2).

**Table 1:** Clinicopathological characteristics of patients undergoing radical gastrectomy

	Total	Pulmonary Complications		
	*N* = 5327	Yes, *n* = 383	No, *n* = 4944	*P*
Age ± SD	59.54 ± 11.24	64.28 ± 10.41	59.17 ± 11.23	<0.001
Gender				0.043
Male	4028 (75.6%)	306 (79.9%)	3722 (75.3%)	
Female	1299 (24.4)	77 (20.1%)	1222 (24.7%)	
Charlson Index				0.001
0–2	5252 (98.6%)	370 (96.6%)	4882 (98.7%)	
≥3	75 (1.4%)	13(3.4%)	62 (1.3%)	
ASA classification				<0.001
1–2	5059 (95.0%)	341 (89.0%)	4718 (95.4%)	
≥3	268 (5.0%)	42 (11.0%)	226 (4.6%)	
Neoadjuvant chemotherapy				0.055
No	5228 (98.1%)	371 (96.9%)	4857 (98.2%)	
Yes	99 (1.9%)	12 (3.1%)	87 (1.8%)	
Tumor diameter ± SD	51.60 ± 27.99	54.63 ± 29.35	51.09 ± 27.72	0.001
TNM stage				<0.001
I	1226 (23.0%)	63 (16.4%)	1163 (23.5%)	
II	986 (18.5%)	56 (14.6%)	930 (18.8%)	
III	3115 (58.5%)	264 (69.0%)	2851 (57.7%)	
Gastrectomy				0.017
Open	2719 (51.0%)	218 (56.9%)	2501 (50.6%)	
Laparoscopic	2608 (49.0%)	165 (43.1%)	2443 (49.4%)	
Extent of resection				<0.001
Total	2945 (55.3%)	250 (65.3%)	2695 (54.5%)	
Distal	2249 (42.2%)	118 (30.8%)	2131 (43.1%)	
Proximal	133 (2.5%)	15 (3.9%)	118 (2.4%)	
Operative time ± SD	221.45 ± 71.72	246.83 ± 89.07	219.513 ± 70.94	<0.001
Blood loss ± SD	152.13 ± 274.15	269.60 ± 751.11	143.08 ± 190.91	<0.001
Adjuvant chemotherapy				<0.001
No	3226 (60.6%)	287 (74.9%)	2939 (59.4%)	
Yes	2101 (39.4%)	96 (25.1%)	2005 (40.6%)	
Surgical period				0.291
1996–2005	1473 (27.7%)	97 (25.3%)	1376 (27.8%)	
2006–2014	3854 (72.3%)	286 (74.7%)	3568 (72.2%)	

**Table 2:** Univariate and multivariate analyses for risk factor of postoperative pneumonia

	Univariate analysis		Multivariate analysis	
	OR (95% CI)	*P*	OR (95% CI)	*P*
Age (for 10‐year increase)	1.520 (1.373–1.683)	<0.001	1.541 (1.386–1.713)	<0.001
Male sex (vs. female sex)	0.766 (0.592–0.992)	0.043	/	
Charlson Index≥3(vs. 0‐2 )	2.767 (1.508–5.077)	0.001	/	
ASA classification≥3(vs.1‐2 )	2.571 (1.817–3.639)	<0.001	2.202 (1.398–2.866)	<0.001
Neoadjuvant chemotherapy (vs. no)	1.806 (0.978–3.332)	0.055	/	
Tumor diameter (for 10 mm increase)	1.103 (1.060–1.147)	<0.001	1.068 (1.024–1.114)	0.002
Tumor stage II (vs. stage I)	1.112 (0.768–1.610)	0.575	/	
Tumor stage III (vs. stage I)	1.709 (1.288–2.269)	<0.001	/	
Open gastrectomy (vs. LG)	1.291 (1.046–1.592)	0.017	1.458 (1.084–1.961)	0.013
Total gastrectomy (vs. distal gastrectomy)	2.296 (1.300–4.053)	0.004	/	
Proximal gastrectomy (vs. distal gastrectomy)	1.675 (1.336–2.100)	<0.001	/	
Operative time (for 30 min increase)	1.145 (1.093–1.201)	<0.001	1.119 (1.048–1.196)	0.001
Blood loss (for 50 mL increase)	1.121 (1.083–1.159)	<0.001	1.105 (1.054–1.158)	<0.001

### Type of complications and their correlation with prognosis

We focus on specific complication's effect on prognoses. The 5‐year survival significantly decreased in patients with postoperative pneumonia (OR = 1.589, *P *< 0.001), while SSIs, postoperative bleeding, intestinal ileus, remnant gastric stasis, chylous leak, and other postoperative complications showed no significant effect on 5‐year survival of gastric cancer patients after surgery (as shown in Data S1).

### Postoperative pneumonia and prognosis

**Figure 1:**
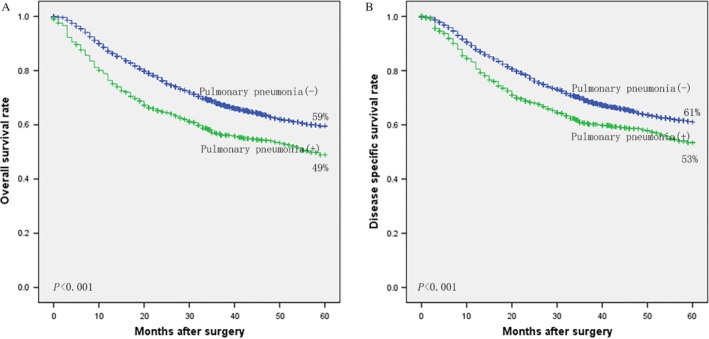
Kaplan–Meier curves of patients with and without of postoperative pneumonia: (A) overall survival, and (B) disease‐specific survival.

**Figure 2:**
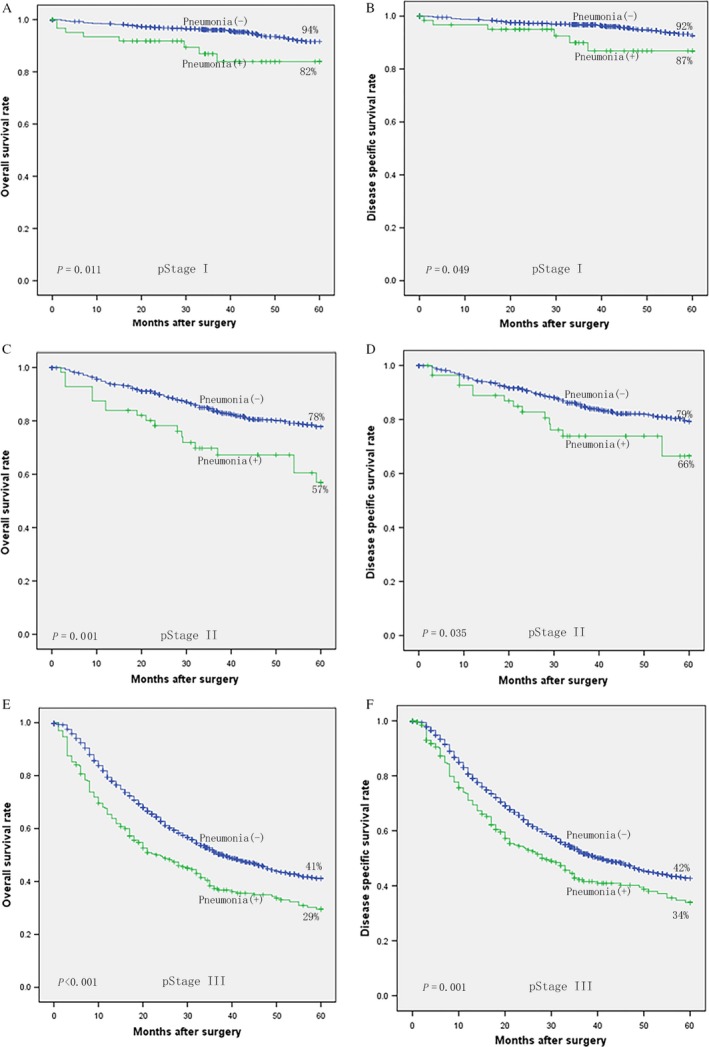
Kaplan–Meier curves of patients with and without of postoperative pneumonia according to pStage: (A) overall survival, (B) disease‐specific survival of pStage I, (C) overall survival, (D) disease‐specific survival of pStage II, (E) overall survival, and (F) disease‐specific survival of pStage III.

Figure 1 demonstrates survival of patients with postoperative pneumonia. The 5‐year overall survival of patients with postoperative pneumonia (*n* = 383) and those without (*n* = 4944) was 49% and 59%, respectively. The difference was significant (log‐rank, *P *< 0.001). The 5‐year specific survival was also lower in patients with postoperative pneumonia than in those without (53% vs. 61%, log‐rank, *P *< 0.001). Further stratified analysis indicated that the reduction in 5‐year overall and specific survival due to postoperative pneumonia existed among all stage I, II, and III patients (log‐rank, *P *< 0.05, as shown in Fig. 2).

### Prognostic factors for gastric cancer patients

Table 3 shows univariate and multivariate analyses of factors affecting the specific survival of the gastric cancer patients. Cox regression analysis suggested that age (HR = 1.117, *P *< 0.001), neoadjuvant chemotherapy (HR = 1.882, *P *< 0.001), tumor size (HR = 1.107, *P *< 0.001), stage II tumor (HR = 2.769, *P *< 0.001), stage III tumor (HR = 8.884, *P *< 0.001), and postoperative pneumonia (HR = 1.259, *P* = 0.006) were independent risk factors for specific survival. Further stratified analyses were performed for each stage of disease, and the results indicated that postoperative pneumonia was independent risk factors for specific survival in stage III patients as shown in Table 4 and Data S3.

**Table 3:** Univariate and multivariate analyses for disease‐specific survival

	Univariate analysis		Multivariate analysis	
	HR (95% CI)	*P*	HR (95% CI)	*P*
Age (for 10‐year increase)	1.125 (1.076–1.176)	<0.001	1.117 (1.067–1.169)	<0.001
Male sex (vs. female sex)	0.979 (0.878–1.091)	0.699	/	
Charlson Index≥3(vs.0–2 )	1.126 (0.758–1.670)	0.557	/	
ASA classification≥3(vs.1–2 )	1.212 (0.997–1.473)	0.054	/	
Neoadjuvant chemotherapy (vs. no)	2.088 (1.557–2.799)	<0.001	1.882 (1.403–2.526)	<0.001
Tumor diameter (for 10 mm increase)	1.237 (1.217–1.258)	<0.001	1.107 (1.085–1.129)	<0.001
Tumor stage II (vs. stage I)	3.301 (2.485–4.385)	<0.001	2.769 (2.078–3.688)	<0.001
Tumor stage III (vs. stage I)	12.394 (9.703–15.831)	<0.001	8.884 (6.878–11.477)	<0.001
Open gastrectomy (vs. LG)	1.401 (1.273–1.543)	<0.001	/	
Total gastrectomy (vs. distal gastrectomy)	1.789 (1.619–1.978)	<0.001	/	
Proximal gastrectomy (vs. distal gastrectomy)	1.195 (0.857–1.667)	0.293	/	
Operative time (for 30 min increase)	1.109 (1.087–1.133)	<0.001	/	
Blood loss (for 50 mL increase)	1.069 (1.052–1.085)	<0.001	/	
Adjuvant chemotherapy (vs. no)	1.149 (1.047–1.261)	0.004	0.885 (0.804–0.973)	0.012
Surgical period 2006–2014 (vs. 1996–2005)	0.988 (0.894–1.092)	0.817	/	
Postoperative pneumonia (vs. no)	1.589 (1.353–1.867)	<0.001	1.259 (1.070–1.483)	0.006
Surgical site infections (vs. no)	1.126 (0.914–1.387)	0.267		

**Table 4:** Univariate and multivariate analyses for disease‐specific survival in stage III

	Univariate analysis		Multivariate analysis	
	HR (95% CI)	*P*	HR (95% CI)	*P*
Age (for 10‐year increase)	1.091 (1.039–1.145)	<0.001	1.069 (1.018–1.568)	0.008
Male sex (vs. female sex)	0.991 (0.883–1.113)	0.881		
Charlson Index ≥3(vs. 0–2 )	1.188 (0.764–1.846)	0.444		
ASA classification ≥3(vs.1–2 )	1.014 (0.818–1.257)	0.900		
Neoadjuvant chemotherapy (vs. no)	1.883 (1.380–2.570)	<0.001	1.663 (1.216–2.274)	0.001
Tumor diameter (for 10 mm increase)	1.102 (1.079–1.125)	<0.001	1.111 (1.088–1.134)	<0.001
Open gastrectomy (vs. LG)	1.007 (0.908–1.118)	0.892		
Total gastrectomy (vs. distal gastrectomy)	1.278 (0.867–1.884)	0.215		
Proximal gastrectomy (vs. distal gastrectomy)	1.266 (1.135–1.411)	<0.001		
Operative time (for 30 min increase)	1.021 (0.998–1.044)	0.078		
Blood loss (for 50 mL increase)	1.010 (0.993–1.027)	0.233		
Adjuvant chemotherapy (vs. no)	0.847 (0.766–0.936)	0.001		
Surgical period 2006–2014 (vs. 1996–2005)	1.369 (1.231–1.522)	<0.001	1.389 (1.246–1.548)	<0.001
Postoperative pneumonia (vs. no)	1.353 (1.139–1.607)	0.001	1.259 (1.057–1.498)	0.010
Surgical site infections (vs. no)	1.152 (0.925–1.435)	0.207		

### Effect of treatment for postoperative pneumonia on prognosis

Incidences and the severity of postoperative pneumonia showed no significant difference between the two periods (*P* = 0.291 and *P* = 0.121, respectively). The postoperative pneumonia cases between 2006 and 2014 were more promptly treated compared to those between 1996 and 2005 (*P *< 0.001) and had shorter hospital stays (*P *< 0.001). The 5‐year disease‐specific survival of those without postoperative pneumonia showed no significant difference between the two periods (61.4% vs. 60.8%, respectively, *P* = 0.646). Meanwhile, 5‐year disease‐specific survival of those with postoperative pneumonia between 2006 and 2014 significantly increased to 57.7% compared to those between 1996 and 2005 (44.2%, *P* = 0.011, OR = 0.725, 95% CI: 0.564–0.930), as shown in Data S2 and S4.

## Discussion

Postoperative pneumonia is a common complication after abdominal surgery, especially radical gastrectomy. The complication may be associated with irritation of the diaphragm during lymphadenectomy and digestive tract reconstruction, traction of the chest wall, inhibition of respiratory and cough reflex center, postoperative incision pain, or long‐term bed rest. Postoperative pneumonia not only threatens the safety of patients after surgery, extends hospital stay, and increases hospitalization cost but also adds an extra burden to the surgical team. The incidence of postoperative pneumonia after surgery for gastric cancer has been reported to be 1.1–12.32%. In our study, the incidence was 7.2% (383/5327), consistent with previous studies. Further studies suggested that postoperative pneumonia was an independent risk factor for prognosis of patients with gastric cancer after surgical resection, especially in stage III.

Recent studies have suggested that postoperative pneumonia and other complications after radical gastrectomy are independent risk factors for long‐term survival of these patients 12, 13, 14, 15. Kubota et al. 13 demonstrated that these complications prolonged postoperative inflammatory response and thus negatively affected the overall and disease‐specific survival of gastric cancer patients after surgical resection. Tokunaga et al. 12 showed that intra‐abdominal infectious complications reduced overall and recurrence‐free survival after radical resection of gastric cancer. Some scholars reported that postoperative pneumonia reduced the long‐term disease‐specific survival of cancer patients 9 and that the effect of postoperative pneumonia on the prognosis of gastric cancer patients after radical surgery has not been reported.

In this study, Kaplan–Meier survival curve analysis showed that postoperative pneumonia affected 5‐year overall and disease‐specific survival of all Stage I, II, and III gastric cancer patients. Cox regression analysis further confirmed that postoperative pneumonia was an independent risk factor for overall and disease‐specific survival of gastric cancer patients after surgical resection. Previous literature has demonstrated that gastric tumors may recur a few years after resection even if a curative R0 according to postoperative pathological diagnosis 26, 27. Complex postoperative recovery processes such as postoperative pneumonia may inhibit the immune response to tumor cell proliferation, leading to reduced disease‐specific survival 9, 10. Further investigations by Goldfarb et al. 28 suggested that inhibition of excessive perioperative catecholamines and prostaglandin response can effectively alleviate immune suppression of the body and can thereby reduce the incidence of tumor recurrence and metastasis. In addition to immune suppression, postoperative pneumonia may also affect the prognosis of gastric cancer patients through other nonimmune factors. For example, postoperative pneumonia may have a great effect on the general condition of the body, leading to reduced disease‐specific and nonspecific survival of these cancer patients. Additionally, Tokunaga et al. 12 demonstrated that abdominal infection and other complications might also affect the long‐term survival of gastric cancer patients through similar mechanisms. Sierzega et al. 29 concluded that anastomotic leak is a prognostic factor after a total gastrectomy, while Roder et al. 30 concluded that the effects of leakage were solely due to increased early mortality. From our data previously 31, we report a total of 3632 gastric cancer patients, and anastomotic leaks were observed in 50 patients (1.4%). In this study, abdominal infectious complications were observed in 185 patients (3.5%), including 59 patients (1.1%) with anastomotic leaks. And we could not determine whether abdominal infectious complication and anastomotic leak were independent prognostic factors for long‐term survival. Also, in this study, surgical site infection, postoperative bleeding, postoperative ileus, remnant gastric stasis, and chylous leak were not found to affect disease‐specific survival.

Except early ambulation and incentive spirometry use, treatment for postoperative pneumonia has shifted to early active intervention during recent years, including bronchoscopic suction, pleural effusion puncture drainage, and early ventilator‐assisted treatment with proper indications. Through active intervention, the healing time of postoperative pneumonia can be significantly shortened, which may lead to milder or reduced duration of immune suppression and thus a better response to tumor cell proliferation and prognosis of the patients.

Compares the postoperative conditions of patients with postoperative pneumonia between different time periods (1996–2005 vs. 2006–2014), incidences, and the severity of postoperative pneumonia showed no significant difference between the two periods, but the postoperative pneumonia cases were more promptly treated, had shorter hospital stays, and higher disease‐specific survival in the last period. It is possible that the increase in prognosis of gastric cancer patients with postoperative pneumonia may be related to improvements in diagnosis and treatment of complications, shortened hospital stays, as well as the changes in treatment concepts for the complications. Additionally, many other important factors, such as tumor stage, adjuvant chemotherapy changed, and proportion of adjuvant chemotherapy received, may have contributed to this improved long‐term outcome.

Our study has some limitations. Firstly, since a retrospective analysis, there were differences in patient's characteristics. The difference could lead to biased estimates of prognoses. Secondly, it is hard to be the culprit when pneumonia cases have other complications, and be the different severity of complication the different impact on prognoses. The date of inflammatory and immune was missed. These results require further verification in multicenter large prospective clinical trials.

In conclusion, postoperative pneumonia after radical gastrectomy is an independent risk factor for prognosis of gastric cancer patients, especially in stage III.

## Conflict of Interest

There are no conflicts of interest or financial ties to disclose from any authors.

## Supporting information


**Data S1.** Effect of complication type on disease‐specific survival.

**Data S2.** Kaplan–Meier curves of patients with and without postoperative pneumonia according to surgical period: disease‐specific survival of (A) surgical period 1996–2005, (B) surgical period 2006–2014, (C) disease‐specific survival of patients without pneumonia, and (D) with pneumonia.

**Data S3.** Univariate and multivariate analyses for disease‐specific survival in stage I/II.

**Data S4.** Postoperative conditions of patients with postoperative pneumonia after radical gastrectomy in different time periods (1996–2005 vs. 2006–2014).
