# Supplementary figures and images for: Prognostic significance of postoperative pneumonia after curative resection for patients with gastric cancer
Source: Cancer Med. 2017 Oct 26;6(12):2757–65. doi: 10.1002/cam4.1163 (PMC5727328; doi:10.1002/cam4.1163)

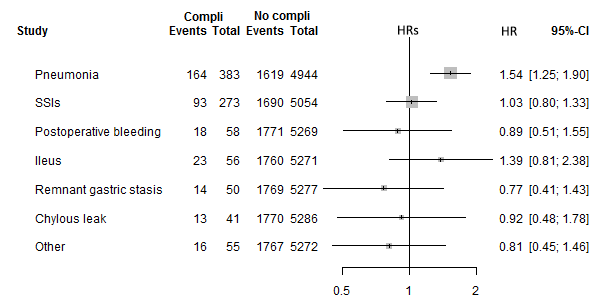

Supplement: Supplementary file 1 — Data S1. Effect of complication type on disease‐specific survival. [file CAM4-6-2757-s001.tif]

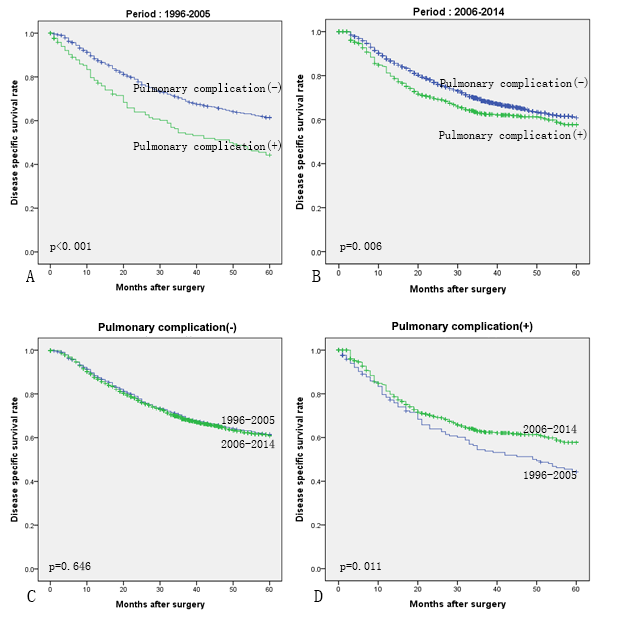

Supplement: Supplementary file 2 — Data S2. Kaplan–Meier curves of patients with and without postoperative pneumonia according to surgical period: disease‐specific survival of (A) surgical period 1996–2005, (B) surgical period 2006–2014, (C) disease‐specific survival of patients without pneumonia, and (D) with pneumonia. [file CAM4-6-2757-s002.tif]
