# Supplementary material for: Prognostic significance of postoperative pneumonia after curative resection for patients with gastric cancer
Source: Cancer Med. 2017 Oct 26;6(12):2757–65. doi: 10.1002/cam4.1163 (PMC5727328; doi:10.1002/cam4.1163)
Supplement: Supplementary file 3 — Data S3. Univariate and multivariate analyses for disease‐specific survival in stage I/II. [file CAM4-6-2757-s003.docx]

Supplement material 3a. Univariate and Multivariate Analyses for Disease-specific Survival in stage I

|  | Univariate Analysis | | Multivariate Analysis | |
| --- | --- | --- | --- | --- |
|  | HR (95% CI) | P | HR (95% CI) | P |
| Age (for 10-year increase) | 1.159（1.245-2.003） | 0.000 | 1.546（1.218-1.963） | 0.000 |
| Male sex (vs female sex) | 0.967（0.551-1.695） | 0.906 |  |  |
| Charlson Index≥3（vs.0-2 ） | 0.985（0.137-7.098） | 0.988 |  |  |
| ASA classification≥3（vs.1-2 ） | 2.253（0.974-5.211） | 0.058 |  |  |
| Neoadjuvant chemotherapy(vs no) | 4.900（1.200-20.015） | 0.027 |  |  |
| Tumor diameter (for 10mm increase) | 1.261（1.129-1.408） | 0.000 | 1.241（1.112-1.385） | 0.000 |
| Open gastrectomy(vs. LG） | 1.117（0.685-1.822） | 0.658 |  |  |
| Total gastrectomy(vs. distal gastrectomy) | 1.051(0.326-3.392） | 0.933 |  |  |
| Proximal gastrectomy(vs. distal gastrectomy) | 1.042(0.622-1.746) | 0.875 |  |  |
| Operative time (for 30 minutes increase) | 1.029（0.923-1.148） | 0.604 |  |  |
| Blood loss (for 50mL increase) | 1.051（0.958-1.154） | 0.292 |  |  |
| Adjuvant chemotherapy(vs no) | 0.869（0.499-1.513） | 0.621 |  |  |
| Surgical period 2006-2014(vs 1996-2005) | 1.088（0.609-1.942） | 0.777 |  |  |
| Postoperative pneumonia (vs no) | 2.318（1.000-5.373） | 0.050 |  |  |
| Surgical site infections (vs no) | 0.669(0.164-2.734) | 0.567 |  |  |

Supplement material 3b. Univariate and Multivariate Analyses for Disease-specific Survival in stage II

|  | Univariate Analysis | | Multivariate Analysis | |
| --- | --- | --- | --- | --- |
|  | HR (95% CI) | P | HR (95% CI) | P |
| Age (for 10-year increase) | 1.329（1.157-1.526） | 0.000 | 1.362（1.184-1.568） | 0.000 |
| Male sex (vs female sex) | 0.775（0.524-1.145） | 0.200 |  |  |
| Charlson Index≥3（vs.0-2 ） | 1.948（0.722-5.255） | 0.188 |  |  |
| ASA classification≥3（vs.1-2 ） | 1.801（1.022-3.173） | 0.042 |  |  |
| Neoadjuvant chemotherapy(vs no) | 1.163（0.371-3.645） | 0.796 |  |  |
| Tumor diameter (for 10mm increase) | 1.097（1.018-1.182） | 0.016 |  |  |
| Open gastrectomy(vs. LG） | 1.626（1.188-2.226） | 0.002 |  |  |
| Total gastrectomy(vs. distal gastrectomy) | 2.451(1.117-5.379） | 0.025 |  |  |
| Proximal gastrectomy(vs. distal gastrectomy) | 1.608(1.161-2.228) | 0.004 |  |  |
| Operative time (for 30 minutes increase) | 1.116（1.042-1.196） | 0.002 | 1.132（1.056-1.213） | 0.000 |
| Blood loss (for 50mL increase) | 1.040（0.983-1.100） | 0.171 |  |  |
| Adjuvant chemotherapy(vs no) | 0.892（0.654-1.218） | 0.474 |  |  |
| Surgical period 2006-2014(vs 1996-2005) | 1.096（0.763-1.574） | 0.619 |  |  |
| Postoperative pneumonia (vs no) | 1.772（1.042-3.013） | 0.035 |  |  |
| Surgical site infections (vs no) | 0.951(0.446-2.027) | 0.896 |  |  |
