# Supplementary material for: Prognostic significance of postoperative pneumonia after curative resection for patients with gastric cancer
Source: Cancer Med. 2017 Oct 26;6(12):2757–65. doi: 10.1002/cam4.1163 (PMC5727328; doi:10.1002/cam4.1163)
Supplement: Supplementary file 4 — Data S4. Postoperative conditions of patients with postoperative pneumonia after radical gastrectomy in different time periods (1996–2005 vs. 2006–2014). [file CAM4-6-2757-s004.docx]

Supplement material 4. Postoperative conditions of patients with postoperative pneumonia after radical gastrectomy in different time periods (1996-2005 vs. 2006-2014).

| Variable | 1996-2005  (n=1464) | 2006-2014  (n=3853) | p |
| --- | --- | --- | --- |
| Postoperative pneumonia | 97 | 286 | 0.291 |
| Clavien–Dindo classification |  |  | 0.121 |
| I-II | 77(79.4%) | 204(71.3%) |  |
| III-IV | 20(20.6%) | 82(28.7%) |  |
| Combine with other complications |  |  | 0.066 |
| No | 18(18.6%) | 80(28.0%) |  |
| Yes | 79(81.4%) | 206(72.0%) |  |
| Postoperative hospital stay±SD | 30.67±21.50 | 22.54±13.10 | 0.000 |
| Treatments |  |  | 0.025 |
| Drugs | 84(86.6%) | 209(73.1%) |  |
| Invasive operations | 6(6.2%) | 37(12.9%) |  |
| Tracheal intubation | 7(7.2%) | 40(14.0%) |  |
